# Evolutionary Simulations Reveal Role for Genomic Recombination in the Evolution of Gene Regulatory Network Complexity and Robustness

**DOI:** 10.1093/gbe/evag196

**Published:** 2026-08-06

**Authors:** Madison Chapel, Carl G de Boer

**Affiliations:** School of Biomedical Engineering, University of British Columbia, Vancouver, Canada; School of Biomedical Engineering, University of British Columbia, Vancouver, Canada

**Keywords:** gene regulatory networks, regulatory complexity, mutational robustness, recombination, evolutionary simulation, gene regulation

## Abstract

The gene regulatory networks of eukaryotes are dramatically more complex than the gene regulatory networks of prokaryotes, but we lack a complete picture of the selective pressures that have shaped this difference. Here, we use a biochemically informed model of gene regulation to simulate gene regulatory network evolution and explore the role that reproductive strategy plays in shaping regulatory complexity. We find that recombining and nonrecombining populations converge to the same level of complexity, even in the absence of selection. However, recombination modifies the rate at which complexity emerges, accelerating convergence to the complexity plateau in changing environments while slowing the process in static environments. Our results suggest that, rather than being under direct selection, regulatory complexity may emerge as a byproduct of other evolutionary processes. These results highlight how reproductive strategy and environmental change interact to influence evolutionary trajectories.

Significance While bacterial genes are known to be regulated by far fewer transcription factors than eukaryotic genes, the reason why these differences in gene regulatory complexity evolved is not known. We used evolutionary simulations to see how the meiotic recombination that is much more typical of eukaryotic reproduction impacted the regulatory complexity, finding that recombination can increase the rate at which complexity emerges but does not impact the final level of complexity. These findings suggest that recombination could be a factor that makes eukaryotic gene regulation more complex.

## Introduction

While many of the mechanisms underlying gene expression are similar in eukaryotes and prokaryotes, the topographies of their gene regulatory networks (GRNs) are fundamentally different (Struhl 1999). Prokaryotes have transcription factors (TFs) that are specific enough to bind only a single site in the genome (Wunderlich and Mirny 2009), and the vast majority of genes are regulated by few TFs, resulting in simple GRNs with few connections between TFs and genes. Meanwhile, the vast majority of eukaryotic TFs are much less specific than prokaryotic TFs. Regulation depends on combinatorial binding by multiple TFs to achieve specificity (Wunderlich and Mirny 2009), resulting in much more complex GRNs with many connections between TFs and genes (Vaishnav et al. 2022).

There are a number of factors that may contribute to the differences in prokaryotic and eukaryotic GRN complexity. One possibility is that these differences are not adaptive, but arose through genetic drift associated with reduced effective population sizes in eukaryotic organisms, as has been proposed for other forms of genomic complexity (Lynch and Conery 2003; Lynch 2007). Supporting this idea, simulations have shown that the gain and loss of TF binding sites is a slow process, particularly for longer motifs, unless selection is strong (as it is in large prokaryotic populations), which could prevent eukaryotes from evolving specific motifs (which are necessarily long) (Tuğrul et al. 2015). However, adaptive explanations have also been proposed. For example, simulations have revealed that weak cooperative interactions between gene products tend to evolve when organisms have to perform a larger number of biological tasks (Gao et al. 2018). As even the simplest eukaryotes have more complex cellular architectures than prokaryotes (Koonin 2010), complex regulatory networks may have evolved to enable the gene expression profiles necessary to complete these tasks. Finally, eukaryotes and prokaryotes differ in the physical organization of DNA within the cell. In eukaryotes, dense chromatin restricts the number of genomic regions accessible to TFs (Guertin and Lis 2013), although this restriction is not sufficient to enable the same degree of specificity as in prokaryotes (Kribelbauer et al. 2019; Perkins et al. 2025).

**Fig. 1.**
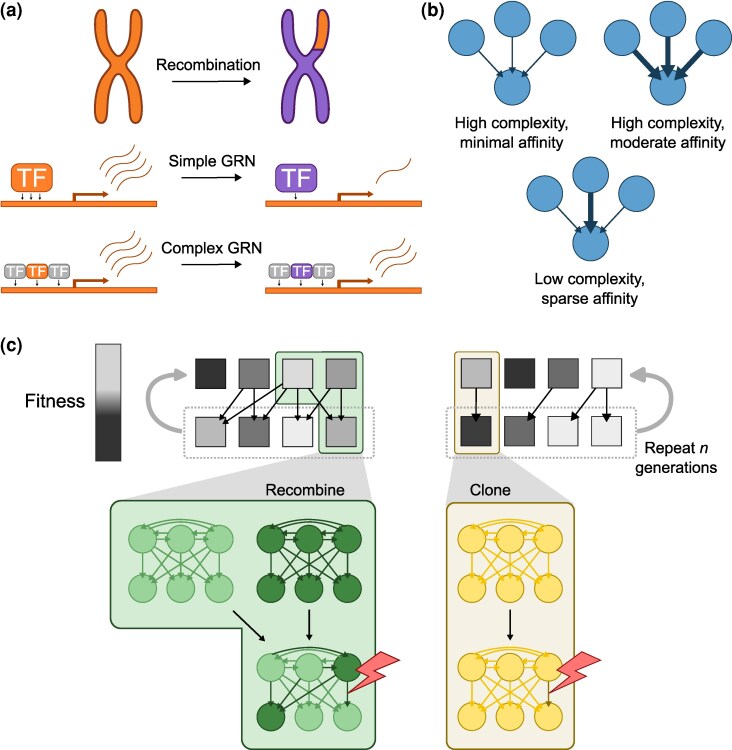
GRN model and simulation setup. a) Recombination can introduce alternate TF alleles (ie alleles from the other parent). In a simple GRN, a differentially acting TF could alter target gene expression more than in a complex GRN, where expression levels would be buffered by the activity of additional TFs. b) GRNs are initialized in one of three states: a high complexity state where each TF binds with minimal affinity (thin arrows), a high complexity state where each TF binds with moderate affinity (heavy arrows), and a low complexity state where a single TF binds with strong affinity while the remainder bind with weak affinity. c) During each generation of the simulation, individuals (squares) are selected with probability proportional to fitness (grayscale intensity). In populations with recombination (green), offspring are a hybrid of two parents (parent A, light shade; parent B, dark shade), inheriting binding affinities (colored arrows) from each. In populations without recombination (yellow), offspring are clones of parents. Offspring in both populations are subject to mutation (lightning bolt) before the process repeats.

One possible explanation for differences in GRN complexity that has been underexplored is how differing reproductive strategies between eukaryotes and prokaryotes may influence the complexity of their regulatory networks. Prokaryotes typically reproduce asexually, resulting in clonal lineages, with mutation as the main driver of genetic variation in the population. While some additional variation can be obtained through horizontal gene transfer, most offspring are nearly identical to their parents. Eukaryotes favor sexual reproduction, with each offspring inheriting a mixture of genes from both parents through recombination. Under asexual reproduction, a tight coupling between TFs and their binding sites could evolve. In contrast, such specificity may be strongly disfavored by a sexual reproductive strategy, as recombination will inevitably combine incompatible TF and binding site alleles. For example, a TF allele from one parent combines with a binding site allele from the other, resulting in dysregulation of the gene in the offspring. In a simple GRN, a differentially acting TF allele would change the expression of its target genes far more than in a complex GRN, where the TF is just one among many contributing to each gene's expression (Fig. 1a).

Previous work has explored the relationship between recombination and robustness, the ability to maintain a stable phenotype after perturbations such as mutation or recombination itself (de Visser et al. 2003). Across numerous studies, recombining populations achieve higher robustness than nonrecombining ones (Azevedo et al. 2006; Misevic et al. 2006; Szöllősi and Derényi 2008; Lohaus et al. 2010; Whitlock et al. 2016; Singhal et al. 2019; Klug and Krug 2022). The underlying mechanisms that drive this increased robustness are yet to be fully understood. Singhal et al. (2019) suggested two paths by which robustness may be achieved: increased physical linkage, so that recombination does not disrupt regulatory interactions, or reduced epistasis, so that loci act more independently. How recombination and regulatory complexity interact to influence robustness remains an outstanding question. We hypothesized that if complexity makes populations more robust to changes introduced during recombination, recombining populations should evolve higher complexity than nonrecombining populations, and more complex GRNs should exhibit greater robustness than less complex ones.

Here, we develop an in silico GRN model and perform evolutionary simulations to explore differences in regulatory complexity and robustness resulting from recombination. By keeping the underlying regulatory architecture constant rather than modeling specific differences between prokaryotic and eukaryotic regulatory networks, we investigate whether recombination alone is enough to drive differences in GRN complexity. We demonstrate that recombining and nonrecombining populations converge to similar complexity levels under a variety of conditions, including mutation rates, initial binding affinity strengths, and starting GRN topologies. However, recombining populations consistently demonstrate greater mutational robustness, indicating that robustness can evolve independently of GRN complexity. Finally, we reveal that, although all populations converge to the same end state of complexity, factors such as recombination and environmental changes influence the rate at which complexity emerges in populations.

## Results

### Using a Biochemically Informed GRN Model to Simulate Evolution

To explore how recombination influences GRN evolution, we developed a simulation framework (Methods). In this simulation, there are two gene types: target genes, the expression of which determines fitness, and TFs, which regulate the target genes and each other, but do not directly impact fitness. The 200 target genes and 20 TFs are randomly arranged on a single linear “chromosome.” Although recombination is typically limited to diploid organisms, all simulations were performed with a haploid model to isolate the effect of recombination without introducing additional confounding variables. Regulatory interactions are encoded in a 20 × 220 matrix of binding affinities, where each entry specifies the strength at which a given TF binds to the promoter region of a given gene.

A gene's expression is the sum of TF binding probabilities (determined by the combined effect of their affinity and expression), each weighted by their effect on expression (+1 for activators, −1 for repressors; *n* = 10 of each). Because there are circular interactions in the regulatory network, expression levels are determined for all genes simultaneously. Expression levels are calculated iteratively until all levels stabilize. If expression levels fail to stabilize, an average is used instead.

Each target gene has a randomly initialized “optimal” expression; its fitness is normally distributed with respect to the log(expression), centered at this optimum. An individual's fitness is the product of the fitness measures for all target genes, simulating a system where all target genes are equal and essential.

In each generation, individuals are selected with probability proportional to their fitness and allowed to reproduce. Without recombination, offspring are a clonal replicate of a parent genome. With recombination, offspring are a hybrid of both parents, with a single randomly selected recombination site determining which genotypes are passed on. The TF affinities for each gene are inherited as a unit from a single parent (ie columns of the binding affinity matrix prior to the recombination site are inherited from one parent, while columns after the recombination site are inherited from the other), simulating inheritance of a single cis-regulatory region for each gene. For both with and without recombination, the TF affinities of offspring are subject to mutation. A new generation then begins, and the process repeats (Fig. 1c). Each simulation was done in 10 replicates with a constant population size of 1,000 across 1 million generations.

### Regulatory Complexity Converges to Similar Plateaus Across Conditions

**Fig. 2.**
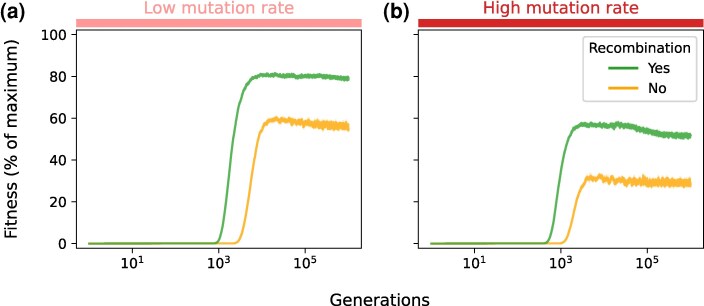
Mutation rate and recombination alter mutation-selection balance. Fitness (*y* axis) shown as mean ± standard error of the mean (SEM; *n* = 10 replicates) for 1 million generations (*x* axis). Recombining (green) and nonrecombining (yellow) populations were evolved with a) low or b) high mutation rates. All populations were initialized with minimal binding affinity.

Using our GRN simulation framework, we first asked how varying the mutation rate impacted GRN evolution. Higher mutation rates would lead to more genetic diversity between individuals, intensifying the selective pressure for greater recombinational robustness and potentially favoring the evolution of more complex GRNs. Higher mutation rates should also increase the mutational burden at each generation, resulting in different fitness trajectories. We tested both high (∼4 per individual per generation) and low (∼1 per individual per generation) mutation rates. Populations with higher mutation rates consistently plateaued at a lower fitness due to the greater mutational burden (Fig. 2), as expected (Haldane 1935). Furthermore, populations reproducing with recombination consistently reached higher fitness levels and evolved more rapidly than those reproducing without recombination, consistent with their increased ability to eliminate deleterious alleles (Fisher 1930; Muller 1932, 1964; Felsenstein 1974).

We next tested how GRN initialization strategy influenced GRN evolution (Methods). Although previous work initialized networks with random affinities (Azevedo et al. 2006), this approach may produce an initial population with substantial fitness variability. Consequently, early generations are dominated by the fittest individuals, resulting in a large “founder effect” and increased variability in simulation outcomes. To avoid this, we initialized GRNs in three different states: two high complexity states, one with minimal initial binding affinities and one with moderate initial binding affinities for all TF–TF and TF–target pairs, and a low complexity state where expression of each TF or target is regulated by a single randomly selected TF with maximal binding affinity while the remaining TFs have minimal affinity (Fig. 1b and Fig. S1). For sparse affinity initialization, the same initial GRN states were used to seed both recombining and nonrecombining populations.

**Fig. 3.**
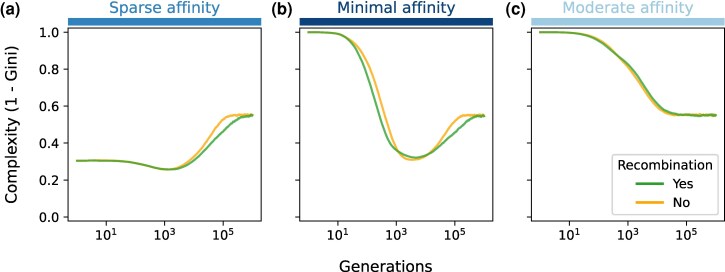
Initial binding affinities alter how complexity emerges but similar plateaus are reached. Complexity (*y* axis) shown as mean ± SEM (*n* = 10 replicates) for 1 million generations (*x* axis). Recombining (green) and nonrecombining (yellow) populations were initialized with a) sparse binding affinity, b) minimal binding affinity, or c) moderate binding affinity. All populations were evolved with high mutation rates.

Despite converging to similar complexity levels (more on this below), different GRN initialization strategies led to distinct evolutionary trajectories. Here, complexity for each gene is quantified as 1 − the Gini index of TF binding probabilities (Vaishnav et al. 2022), with binding probabilities determined by binding affinity and TF expression (Granek and Clarke 2005). GRN complexity is then calculated as the mean complexity across all genes (Methods). Populations initialized in the low complexity state, where each gene is strongly bound by only a single TF, initially maintained low complexity before gradually increasing as additional regulatory interactions accumulate (Fig. 3a). A similar trend was observed in populations initialized with minimal affinity GRNs (Fig. 3b). Although these began with maximal complexity due to equal input from all TFs, a low complexity state rapidly emerges as strong binding affinities become fixed in the population. From here, complexity increases similarly to the populations initialized in the low complexity state.

In contrast, the populations initialized with moderate binding affinities followed a slightly different trajectory. Here, all activating and repressing TFs contribute moderately and equally to expression at the beginning of the simulation. Adaptive mutations are partially buffered by other regulating TFs, resulting in a gradual reduction in complexity as some edges are strengthened and others are pruned (Fig. 3c). This buffering may reduce the individual impact of mutations, resulting in smaller fitness differences between individuals, and causing populations to reach the fitness plateau more slowly than populations initialized with minimal or sparse affinities (Fig. S3). Interestingly, while GRN complexity converged to a similar plateau regardless of GRN initialization, convergence occurred more rapidly in populations without recombination (Fig. 3 and Fig. S4).

### Changing Environments Increase the Rate at Which Complexity Plateaus

In our previous simulations, expression goals remained constant, allowing populations to optimize their regulatory networks under stable conditions. However, selective pressures are rarely static—environments change over time, populations migrate, and other organisms create competition. Any of these scenarios could change expression optima. We reasoned that a changing environment might result in increased regulatory complexity due to the presence of lingering regulatory interactions from past adaptations. The new expression optimum could be achieved by novel regulatory interactions that counteract existing ones.

Additionally, complex regulatory networks may facilitate faster adaptation to shifting conditions through polygenic adaptation (Pritchard et al. 2010; Milligan et al. 2025). In a simple GRN, where a target gene is regulated by a single TF, matching a new optimum typically requires the fixation of a large-effect mutation, such as one altering the binding of a TF or one that alters expression of a regulating TF. In contrast, when a gene is influenced by multiple TFs, with each contributing relatively little, selection can act on many loci simultaneously and adaptation can proceed through small changes throughout the network that combine to have greater effects. Recombining populations are particularly well-suited to this strategy, as they can combine independently beneficial variants that may already exist as standing variation within the population (Fisher 1930; Muller 1932, 1964; Felsenstein 1974). Accordingly, we next tested whether environmental shifts could promote the evolution of regulatory complexity.

**Fig. 4.**
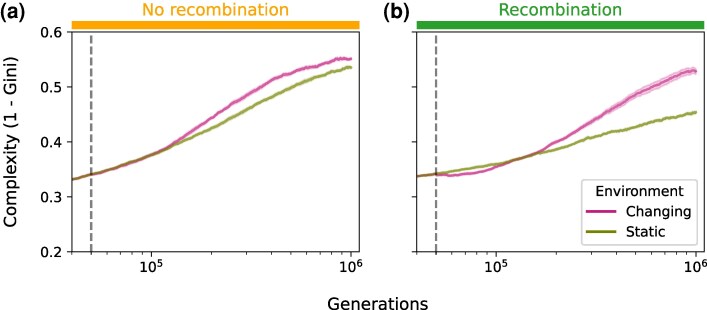
Complexity emerges more rapidly during environmental changes, especially for recombining populations. Complexity (*y* axis) shown as mean ± SEM (*n* = 10 replicates) up to 1 million generations (*x* axis) for populations under static (olive) or changing (pink) environmental conditions. Populations were evolved either a) without recombination or b) with recombination. All populations were initialized with minimal binding affinities and evolved with low mutation rates. Environmental changes begin at the generations marked by vertical dashed lines.

We altered our simulation by introducing environmental shifts after populations have adapted to their initial environment, beginning at 50,000 generations. From then on, the expression optima change for a subset of 20 randomly selected target genes every time the population approaches the fitness plateau (Fig. S5). Because populations initialized with minimal binding affinities quickly evolve through a sparse state, these simulations were only performed in populations initialized with minimal (Fig. 4) or moderate binding affinities (Figs. S6 and S7).

As expected, recombining populations adapted to new environments faster than nonrecombining ones, requiring an average of 1,780 and 2,623 generations, respectively, to regain fitness after each shift (Fig. S8). Despite repeated environmental changes, regulatory complexity ultimately plateaued at a similar level across all conditions, stabilizing at around ∼0.55—the same value observed previously in the static environments. However, for populations that had not yet reached this level when environmental shifts were introduced, the changing environment accelerated the rate at which complexity reached the plateau. This was particularly pronounced in recombining populations (Fig. 4b and Figs. S7 and S8).

We performed an additional set of simulations with a delayed onset of environmental changes, allowing populations' fitnesses to converge in their initial environments. For populations with high mutation rates, environmental changes began at 150,000 generations, while populations with low mutation rates were given 300,000 generations to adapt to their initial environments. These additional simulations produced similar results, with changing environments accelerating convergence toward the complexity plateau, suggesting that this behavior is robust to environmental changes beginning at different timepoints (Figs. S9 and S10).

### Recombining Populations Achieve Higher Robustness

**Fig. 5.**
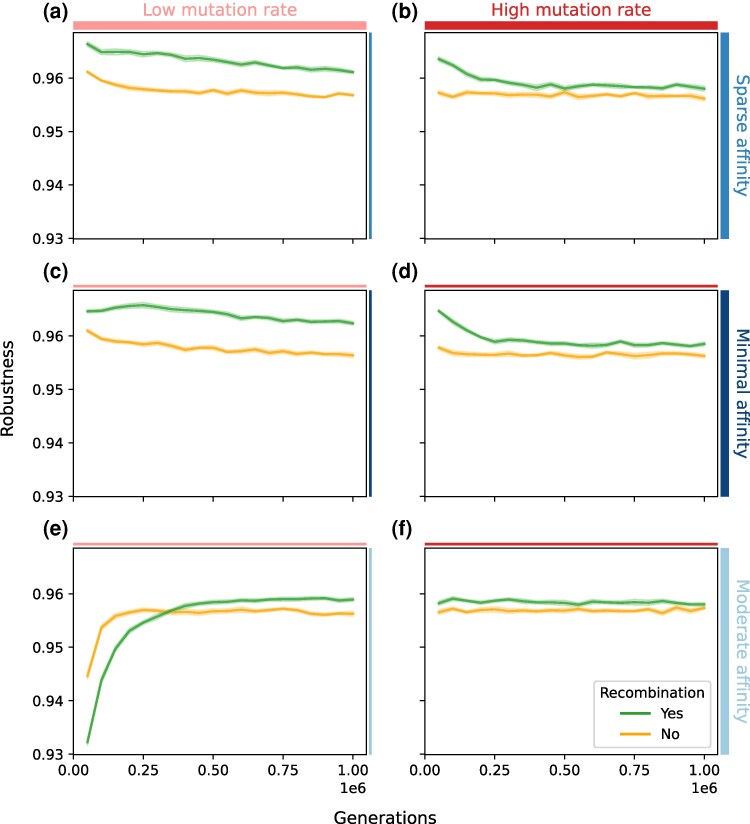
Recombining populations consistently evolve higher robustness than nonrecombining. Mutational robustness (*y* axis) shown as mean ± SEM (*n* = 10 replicates) for 1 million generations (*x* axis). Populations were evolved with (green) or without (yellow) recombination. Panels show: a, c, e) low mutation rate, b, d, f) high mutation rate, a, b) sparse initial affinity, c, d) minimal initial affinity, e, f) moderate initial affinity.

We next investigated whether recombination increased robustness, and whether mutational robustness increased with increasing regulatory complexity. To test this, we perturbed individual GRNs by introducing mutations. “Robustness” was measured by determining the mean Euclidean distance between the original expression profile and 100 mutated profiles (Methods). Despite converging at similar complexity levels, recombining populations consistently evolved greater mutational robustness than nonrecombining ones (Fig. 5 and Fig. S11), as demonstrated in previous studies (Azevedo et al. 2006; Misevic et al. 2006; Szöllősi and Derényi 2008; Lohaus et al. 2010; Whitlock et al. 2016; Singhal et al. 2019; Klug and Krug 2022). Furthermore, no correlation was found between complexity and robustness (Pearson's *r*^2^ < 0.014 for all populations tested; Fig. S12). The trend of greater robustness in recombining populations was observed regardless of mutation rate or initialization conditions, although these variables did affect the robustness in different ways, especially at earlier generations (Fig. 5). A similar effect was observed, though with increased noise, when testing mutational robustness in changing populations (Fig. S11). The noisier results here likely result from the different replicate populations being at different stages of adaptation to new environments at each time point, since robustness is measured every 50,000 generations regardless of when the population last experienced an environmental shift.

### Similar Regulatory Complexity Is Produced by Neutrally Evolving Populations

To determine whether the consistently observed complexity plateau represents an adaptive optimum or a neutral byproduct, we evolved populations without selection. Instead of selecting individuals with probability proportional to their fitness, individuals were selected randomly at each generation. All other parameters (ie mutation rate, initial binding affinities, and recombination) were matched to previous simulations. These simulations represent an evolutionary null, providing an expectation of regulatory complexity in the absence of selection.

**Fig. 6.**
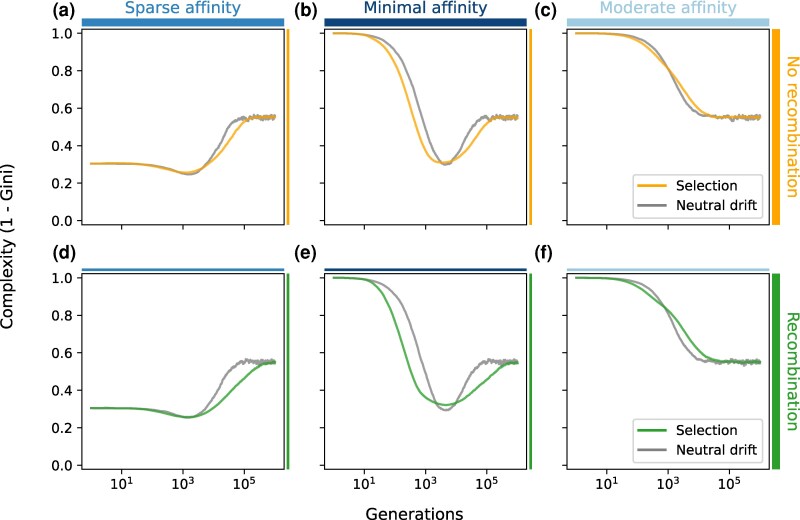
Complexity plateaus more rapidly in neutrally evolving populations. Complexity (*y* axis) shown as mean ± SEM (*n* = 10 replicates) for 1 million generations (*x* axis). Populations were evolved with (colors) or without (gray) selection. Panels show: a, d) sparse initial affinity, b, e) minimal initial affinity, c, f) moderate initial affinity, a–c) no recombination, d–f) recombination. All populations were evolved with high mutation rates.

While the neutrally evolving populations converge to the same complexity as matched populations under selection, they did so more rapidly than with selection (Fig. 6). In static environments, selection thus appears to delay this convergence, an effect that is more pronounced when recombination is present.

## Discussion

In our simulations, recombining populations consistently evolved GRNs that were more robust to mutational perturbation. This finding aligns with previous work demonstrating smaller mutational effects in recombining finite populations (Azevedo et al. 2006; Misevic et al. 2006; Szöllősi and Derényi 2008; Lohaus et al. 2010; Whitlock et al. 2016; Singhal et al. 2019; Klug and Krug 2022). Our simulations also captured the widely demonstrated effect of recombining populations adapting to new environments faster than nonrecombining ones (Malmberg 1977; Greig et al. 1998; Rice and Chippindale 2001; Colegrave 2002; Poon and Chao 2004; Goddard et al. 2005; Becks and Agrawal 2012; Luijckx et al. 2017).

Our simulations suggest that recombination and changing environmental pressures can influence the emergence of regulatory complexity. Reproductive mode alone did not drive the evolution of more complex GRNs, as recombining and nonrecombining populations tended to plateau at similar levels of regulatory complexity. Even when selection was removed entirely, complexity still reached the same plateau. However, recombination did influence the rate at which complexity plateaued. Populations under selection plateaued more slowly than neutrally evolving populations, with this effect being most pronounced in recombining populations. When the environment was dynamic, recombination drove populations to the plateau more rapidly than had been observed in static environments. Nonrecombining populations also approached this plateau more rapidly in changing environments than in static ones, though the difference was less dramatic than the recombining populations.

In static environments, when populations are near the fitness optimum, most mutations will be neutral or deleterious. Recombination enables deleterious alleles that occur in otherwise fit backgrounds to be eliminated because they can be separated from beneficial alleles. This allows recombining populations to maintain a more stable phenotype but also limits the population's ability to navigate the space of viable regulatory networks, slowing the convergence of regulatory complexity compared with nonrecombining populations. In contrast, when selection pressures change, recombination promotes rapid adaptation by combining beneficial variants from otherwise unfit backgrounds (McDonald et al. 2016; Pai et al. 2026). In simulations under selection, complexity emerged most rapidly for recombining populations in changing environmental conditions, suggesting that when selection pressures shift, recombination instead promotes exploration of genotype space. Thus, the changes in complexity may reflect changes in how recombination mediates adaptation rather than selection for complexity.

Consistent with previous simulations of gene regulatory evolution, there may be minimal selection on complexity, and instead the complexity of extant regulatory sequences may simply reflect sampling from the set of all GRNs capable of satisfying the gene expression constraints, ie the solution space (He et al. 2012; Vaishnav et al. 2022). The dimensionality of this solution space gives insight into why populations under selection and neutrally evolving populations converge to similar levels of complexity. Although selection constrains gene expression, our simulations place no constraints on how individual TFs regulate target genes. Because many TFs contribute to the regulation of each gene, there are numerous combinations of regulatory interactions capable of producing similar expression levels. Consequently, selection may reduce the space of viable GRNs very little relative to a neutrally evolving population, and populations will continue to explore the space of equivalently-fit regulatory states. Since the space of expression-satisfying GRNs is vast, evolution will tend to produce solutions in accordance with how often they occur, which can resemble those that arise in neutrally evolved populations.

Maintaining specific regulatory interactions can be difficult in cases where TFs are highly interchangeable: loss of binding by one TF can often be compensated for by strengthening the contribution of another. Consequently, regulatory interactions may continue to drift even while expression remains near the fitness optimum. Rather than a narrow fitness peak containing only a small number of viable GRNs, there is instead a broad, high-dimensional fitness plateau composed of many similarly fit regulatory configurations. This interpretation also helps explain why recombination alters the rate at which complexity emerges without substantially changing the final plateau itself. However, in biological systems, TFs are not always interchangeable, such as when they respond directly to environmental stimuli (Shepherd et al. 2023), necessitating that certain regulatory interactions are maintained.

Instead of providing adaptive benefit, the different complexities of prokaryote and eukaryote GRNs could simply reflect the different gene regulatory hardware they utilize. While we simulated recombining and nonrecombining populations with identical hardware, the reality is that prokaryotic TFs tend to be far more specific than eukaryotic TFs (Wunderlich and Mirny 2009). The question then becomes, why do prokaryotes have such specific TFs relative to eukaryotes? Future work should aim to refine GRN models with additional regulatory mechanisms, such as environmental responsiveness, to more accurately reflect biological systems.

While our GRN model draws on established literature to simulate regulatory networks in a biologically informed manner (Granek and Clarke 2005; Vaishnav et al. 2022), several simplifications were made to streamline the design and improve the model's interpretability. Our model uses a single linear chromosome with one recombination point per generation, while most natural populations undergoing recombination have multiple chromosomes that each recombine separately, increasing their abilities to separate beneficial and deleterious alleles. In a recombining model with multiple chromosomes, we would expect to see complexity converge even more slowly in static environments than we observed in our simulations and converge even faster in changing environments. Similarly, while we used a haploid model, we would expect this effect to be enhanced in a diploid organism due to a greater genetic diversity present in the population and the independent assortment of homologous chromosomes, which further increases the efficiency with which sexual reproduction separates deleterious and beneficial mutations.

In our model, TFs functioned exclusively as activators or repressors. In reality, TFs can have both activating and repressing activity in different contexts, can be conditionally active only after receiving an appropriate signal (Bondra and Rine 2023), and these activities can change over evolutionary time. Furthermore, the number of TF and target genes was fixed, preventing regulatory networks from evolving via gene duplication—a process partially responsible for the redundancy in eukaryotic binding motifs (Rosanova et al. 2017). Mutations in TF coding sequences were not simulated here. While they tend to be uncommon relative to cis-regulatory mutations (Weirauch and Hughes 2010), TF coding mutations could have even more widespread effects, altering the activity of that TF throughout the genome. To make our simulations computationally tractable and to avoid introducing additional sources of variability, we tested only a single, constant, population size of 1,000 individuals. Future simulations may explore whether drift in small populations or bottlenecks of reduced population size influence the emergence of regulatory complexity.

## Methods

### Modeling GRNs

#### Binding Affinity Matrix

Individual GRNs were modeled as a matrix of binding affinities bounded between ln(*k*) = −5.0 and ln(*k*) = 5.0, with 20 TFs and 200 target genes. Half of the TFs were designated as activators and half as repressors. All TFs were equally strong repressors or activators once bound. TF and target genes were randomly distributed throughout a single linear chromosome. TFs regulate the expression of all target genes and each other, resulting in a 20 × 220 matrix whose elements ln(*k_i,j_*) describe the binding affinity of the TF product of gene *i* to the promoter of gene *j*.

#### Gene Expression

The binding probability *R_i,j_* of TF *i* to gene *j* was adapted from Granek and Clarke (2005):


$$R_{i,j}=\frac{[TF_{i}]k_{i,j}}{1+[TF_{i}]k_{i,j}},$$

where [TF*_i_*] is the expression level of TF*_i_* and *k_i_*_,*j*_ is the binding affinity of TF*_i_* to gene *j*.

The total regulatory activity *R_j_* of all TFs acting on gene *j* was calculated as the sum of the binding probabilities of all activators minus the sum of the binding probabilities for all repressors:


$$R_{j}=\;\underset{i\in Act}{\sum }\;R_{i,j}-\underset{i\in Rep}{\sum }\;R_{i,j},$$

where *Act* is the set of all activating TFs while *Rep* is the set of repressing TFs. The regulatory activity was then passed into a sigmoidal function to determine the expression level *E* in log space:


$$\ln\;(E_{j})=\;\left(\frac{1}{1+e^{-R_{j}}}-0.5\right)\cdot 2.$$

Expression was calculated iteratively until expression levels for all genes had stabilized (differences of <1 × 10^−4^ between iterations). If expression levels failed to stabilize within 5,000 iterations, an average of the expression levels over all iterations was used. For the first iteration, log expression levels were randomly sampled uniformly between −1 and 1, with the same initialization applied to every individual in the population.

Each of the 200 target genes was assigned an optimal expression level, $E_{j}^{opt}$. These values were drawn from a uniform distribution in log space:


$$E_{j}^{opt}=e^{x},\;\;\;\mathrm{where}\;\;x∼U(-1,1).$$

#### Fitness

The fitness of an individual, *F*, was calculated as the product of the fitness of all target genes. The fitness of each target gene was defined by how closely its expression level, *E_j_*, matched the optimal expression level, $E_{j}^{opt}$, using a Gaussian function with a standard deviation of *σ* = 0.75. While genes in real biological systems may have different fitness functions (ie narrower or wider fitness optimal expression levels, step-wise functions; Keren et al. 2016), a single fitness function was used to avoid introducing additional sources of variability. The standard deviation of the fitness function acts as a modifier for selection strength. Larger values result in a wider range of expression levels being tolerated, increasing the maximum fitness that populations can achieve. To confirm that the selected value would not influence complexity, we tested three different values (Figs. S13 and S14). Expression levels were compared in log space:


$$F=\;\prod \frac{e^{(-{\left(\ln\;(E_{j})-\ln\;(E_{j}^{opt})\right)}^{2})/2\sigma^{2}}}{\sigma \sqrt{2\pi }}.$$

#### Complexity

Complexity of a GRN was measured using the Gini coefficient (Vaishnav et al. 2022). While typically used in economics to quantify inequalities in wealth distribution, the Gini coefficient can also model other inequalities within a population, such as the degree of inequality in regulatory interactions of TFs in a regulatory network. A Gini coefficient of 0 represents a distribution where all members of the population have equal wealth, while a coefficient of 1 represents a distribution where all wealth is controlled by a single individual. Complexity of regulatory interactions is measured as 1 − Gini, and ranges from 0 (expression is controlled by a single TF) to 1 (all TFs contribute equally to expression). Complexity, *C*, for each gene was measured using the binding probabilities, *R*, sorted in ascending order, of *n* TFs acting on it:


$$C_{j}=1-\;\frac{\sum_{i=1}^{n}\;(2i-n-1)R_{i,j}}{n\sum_{i=1}^{n}\;R_{i,j}}.$$

Complexity for each individual was measured as the mean complexity of all genes.

#### Robustness

Robustness was measured by introducing mutations to a GRN and measuring changes in expression. Mutation effect sizes were sampled from a Gaussian distribution with standard deviation of 2.0 and applied to the matrix of binding affinities, following the same procedure for mutations during evolution simulations (see below). The change in expression was measured as the Euclidean distance between the vector of initial expression pattern and the expression pattern after mutation, compared in log space. Robustness, *ρ*, was measured every 50,000 generations across *k* = 100 mutations:


$$\rho =1-\;\frac{1}{100}\overset{100}{\underset{k=1}{\sum }}\;\sqrt{\overset{m}{\underset{\;j=1}{\sum }}\;{\left(\ln\;(E_{j})\;-\;\ln\;(E_{j}^{opt})\right)}^{2}}.$$

### Evolution Simulations

Evolution was simulated using a genetic algorithm (GA) implemented with the distributed evolutionary algorithms in Python package (Fortin et al. 2012). For all simulations, the population size was 1,000 and reproduction probability was 0.5. GRNs within a population were initialized in one of three states: a high complexity state with minimal initial binding affinities for all TF–TF and TF–target pairs, a high complexity state with moderate initial binding affinities, and a low complexity state with sparse affinities where expression of each TF or target is regulated by a single randomly elected TF with maximal binding affinity while the remaining TFs have minimal affinity (Fig. S1).

The moderate binding affinity (*k*) was defined as ln(*k*) = 0.0. For the minimal affinity initialization, our goal was to evolve GRNs from a state without regulatory interactions. However, because affinities were represented in log space, it was necessary to determine a sufficiently low cut-off for a minimal binding affinity value. A value of 0 (no affinity) cannot be represented in log space, and choosing too low a value would result in GRNs evolving slowly or not at all (eg a mutation from ln(*k*) = −10.0 to ln(*k*) = −9.0 may not alter expression enough to confer a fitness advantage). The minimal initial binding affinity was set such that initial expression values varied minimally from the basal expression level of ln(*E*) = 0.0. To achieve this, we tested a range of binding affinities and selected the largest value that yielded expression levels with a standard deviation ≤0.025 in log space. This value depended on the number of TFs in the network and was determined to be ln(*k*) = −(0.517 · ln(*n*) + 2.516), where *n* is the number of TFs (in our case of 20 TFs, ln(*k*) = −4.06) (Fig. S2). For the high complexity initialization, a single maximal initial binding affinity was set as ln(*k*) = (0.517 · ln(*n*) + 2.516), which, with 20 TFs, is ln(*k*) = 4.06.

For populations under selection, selection was performed using a roulette function, where the probability of selection is proportional to fitness. For neutrally evolving populations, selection was performed randomly where each individual had equal probability of being selected.

For recombining populations, a custom one-point crossover function was used to independently generate two distinct recombinant offspring for each mating pair. Each offspring inherited all binding affinities prior to a randomly selected crossing-over point from one parent and all binding affinities after the crossover point from the other parent. The process was repeated with a new crossover point to generate the second offspring.

Mutation effect sizes were sampled from a Gaussian distribution with standard deviation of 2.0, designed to enable minimal or maximal expression to be achieved within two mutations, mimicking the plasticity previously observed for yeast promoters measured in a reporter system (Vaishnav et al. 2022). Mutation effects were applied to the binding affinities (*k_i_*_,*j*_) *in* logarithmic space. Mutation effect sizes, similar to the fitness function, act as a modifier for selection strength. A larger mutation effect size results in a lower terminal fitness due to the higher per-generation mutation burden, but faster adaptation because similar changes in fitness can be acquired through fewer mutations. To verify that these parameter choices would have minimal influence on complexity, we simulated a range of values (Figs. S15 and S16). The mutation probability was set such that each new individual would receive an expected 4.4 (*P* = 1.0 × 10^−3^, high mutation rate) or 1 (*P* = 2.2727 × 10^−4^, low mutation rate) mutations. While these are higher than typical per-base mutation probabilities in biological systems, lower mutation rates resulted populations failing to reach a complexity equilibrium within a feasible simulation timescale.

Each GA was run for 1 million generations. A total of 10 replicate populations were evolved for each set of conditions. Optimal expression goals were randomly initialized for each replicate and matched across populations with or without recombination.

To simulate selection in changing environmental conditions, simulations were resumed at 50,000 generations, after fitness levels had plateaued. The mean fitness of the population was determined as a percentage of the maximum possible fitness. Twenty target genes (10% of the 200 target genes) were selected at random, and their optimal expression values shifted by values sampled from a Gaussian distribution with standard deviation of 0.25, simulating a change in the environment and selection for a new expression pattern. The number of genes was chosen to represent an environmental change impacting a substantial fraction of target genes. The magnitude of expression shifts was calibrated to be sufficiently large that a clear decline in fitness would occur, but also small enough that populations could recover within a few thousand generations, allowing many environmental shifts to be simulated. New optimal expression values were bounded between ln(−0.9) and ln(0.9) to prevent the emergence of difficult to attain expression goals (eg requiring maximum expression for all target genes). To prevent a cumulative decline in fitness, environmental shifts were repeated only when the population's mean fitness was within a percentage point of the mean fitness at the time at which environmental shifts began. During every environmental shift, a new subset of 20 target genes was selected.

## Supplementary Material

evag196_Supplementary_Data

## Data Availability

Code used to run simulations and analyze results can be found at https://github.com/de-Boer-Lab/GRN-evolution/.
