# Supplementary material for: Evolutionary Simulations Reveal Role for Genomic Recombination in the Evolution of Gene Regulatory Network Complexity and Robustness
Source: Genome Biol Evol. 2026 Aug 6;18(8):evag196. doi: 10.1093/gbe/evag196 (PMC13455014; doi:10.1093/gbe/evag196)
Supplement: Supplementary file 1 [file evag196_supplementary_data.docx]

Evolutionary simulations reveal role for genomic recombination in the evolution of gene regulatory network complexity and robustness

Supplementary Material

Madison Chapel^1^, Carl G de Boer^1*^

^1^School of Biomedical Engineering, University of British Columbia, Vancouver, Canada

^*^Corresponding author: carl.deboer@ubc.ca

Supplementary Figures

**Supplementary Figure S1. Sample GRNs in various states.** GRNs are represented as a matrix of log binding affinities (colour) for 20 TFs (y-axis) regulating all other TFs and target genes (x-axis). Shown here are GRN log binding affinities (color) initialized with minimal, moderate, and sparse binding affinities, and a GRN that has converged at the complexity plateau. When initialized with sparse binding affinity, a single TF per column binds with maximal affinity while the remainder bind with minimal affinity.

**Supplementary Figure S2. Determining minimal binding affinity initialization. (A)** Relationship between sufficiently low binding affinity (ln(*k*)) values (y-axis) and number of TFs in a network (x-axis). A regression line was fit to cut-off values (points) for varying numbers of TFs. **(B)** Distributions of gene expression values (x-axis) for a network with 20 TFs initialized with varying binding affinities. **(C)** Relationship between binding affinity strength (x-axis) and standard deviation of expression (y-axis) for a network of 20 TFs. The threshold of σ = 0.025 is indicated with a dashed line, and the largest binding affinity value (ln(*k*) = -4.10) not exceeding the threshold is indicated in orange.

**Supplementary Figure S3**. **Fitness trajectories for all simulations in static environments.** Fitness (y-axis) shown as mean ± SEM (n = 10 replicates) for 1 million generations (x-axis). Populations were evolved with (green) or without (yellow) recombination. Panels show: (**A, C, E)** Low mutation rate, **(B, D, E)** high mutation rate, **(A, B)** sparse initial affinity, **(C, D)** minimal initial affinity, **(E, F)** moderate initial affinity.

**Supplementary Figure S4**. **Complexity trajectories for all simulations in static environments.** Complexity (y-axis) shown as mean ± SEM (n = 10 replicates) for 1 million generations (x-axis). Populations were evolved with (green) or without (yellow) recombination. Panels show: **(A, C, E)** Low mutation rate, **(B, D, E)** high mutation rate, **(A, B)** sparse initial affinity, **(C, D)** minimal initial affinity, **(E, F)** moderate initial affinity.

**Supplementary Figure S5**. **Environmental changes cause fitness to drop and recover.** Example of average fitness (y-axis) evolution for a single replicate population during environmental changes, beginning at generation (x-axis) 50,000. Population was initialized with moderate binding affinity and evolved with recombination and a high mutation rate.

**Supplementary Figure S6. Non-recombining populations: changing environments accelerate complexity convergence unless plateau is already reached.** Complexity (y-axis) shown as mean ± SEM (n = 10 replicates) up to 1 million generations (x-axis) under static (olive) or changing (pink) environments. Panels show: (A, C) Low mutation rate, (B, D) high mutation rate, (A, B) minimal initial affinity, (C, D) moderate initial affinity. Environmental changes begin at 50,000 generations, indicated by the vertical dashed lines.

**Supplementary Figure S7. Recombining populations: changing environments accelerate complexity convergence unless plateau is already reached.** Complexity (y-axis) shown as mean ± SEM (n = 10 replicates) up to 1 million generations (x-axis) under static (olive) or changing (pink) environments. Panels show: (A, C) Low mutation rate, (B, D) high mutation rate, (A, B) minimal initial affinity, (C, D) moderate initial affinity. Environmental changes begin at 50,000 generations, indicated by the vertical dashed lines.

**Supplementary Figure S8. Recombining populations recover more quickly after environmental changes.** Mean generations between environment changes (y-axis) for recombining (green) and non-recombining (yellow) populations across different test conditions (x-axis).

**Supplementary Figure S9. Non-recombining populations: changing environments accelerating complexity convergence is robust to different lengths of initial adaptation in static environments.** Complexity (y-axis) shown as mean ± SEM (n = 10 replicates) up to 1 million generations (x-axis) under static (olive) or changing (pink) environments. Panels show: (A, C) Low mutation rate, (B, D) high mutation rate, (A, B) minimal initial affinity, (C, D) moderate initial affinity. Environmental changes begin at 150,000 generations (high mutation rate) or 300,000 generations (low mutation rate), indicated by the vertical dashed lines.

**Supplementary Figure S10. Recombining populations: changing environments accelerating complexity convergence is robust to different lengths of initial adaptation in static environments.** Complexity (y-axis) shown as mean ± SEM (n = 10 replicates) up to 1 million generations (x-axis) under static (olive) or changing (pink) environments. Panels show: (A, C) Low mutation rate, (B, D) high mutation rate, (A, B) minimal initial affinity, (C, D) moderate initial affinity. Environmental changes begin at 150,000 generations (high mutation rate) or 300,000 generations (low mutation rate), indicated by the vertical dashed lines.

**Supplementary Figure S11. Recombining populations tend to evolve higher robustness than non-recombining populations in changing environments.** Mutational robustness (y-axis) shown as mean ± SEM (n = 10 replicates) for 1 million generations (x-axis). Populations were evolved with (green) or without (yellow) recombination. Panels show: (A, C) Low mutation rate, (B, D) high mutation rate, (A, B) minimal initial affinity, (C, D) moderate initial affinity. Environmental changes begin at the generations marked by vertical dashed lines.

**Supplementary Figure S12. Complexity and robustness are uncorrelated.** Pearson's *r*^2^ values (y-axis) between complexity and robustness across all test conditions (x-axis), calculated individually for each population of 1,000 individuals (dots). Correlation was tested every 50,000 generations for each of 10 replicate populations per condition (n = 200 tests).

**Supplementary Figure S13. Fitness trajectories for simulations with modified fitness functions.** Fitness (y-axis) shown as mean ± SEM (n = 10). Populations were evolved with fitness assessed according to a Gaussian function with varying distributions: σ = 0.5 (red), σ = 0.75 (black; value used in main simulations), and σ = 1.0 (blue). Panels show: **(A, C, E)** No recombination, **(B, D, F)** recombination, **(A, B)** sparse initial affinity, **(C, D)** minimal initial affinity, **(E, F)** moderate initial affinity. All populations were evolved with high mutation rates.

**Supplementary Figure S14. Complexity trajectories for simulations with modified fitness functions.** Complexity (y-axis) shown as mean ± SEM (n = 10 replicates). Populations were evolved with mutation effect sizes sampled from varying Gaussian distributions; σ = 0.5 (blue), σ = 0.75 (black; value used in main simulations) and σ = 1.0 (red). Panels show: **(A, C, E)** No recombination, **(B, D, F)** recombination, **(A, B)** sparse initial affinity, **(C, D)** minimal initial affinity, **(E, F)** moderate initial affinity. All populations were evolved with high mutation rates.

**Supplementary Figure S15. Fitness trajectories for simulations with modified mutation effect distributions.** Fitness (y-axis) shown as mean ± SEM (n = 10 replicates). Populations were evolved with mutation effect sizes sampled from varying Gaussian distributions; σ = 0.5 (teal), σ = 1.0 (blue), σ = 2.00 (black; value used in main simulations) and σ = 4.0 (red). Panels show: **(A, C, E)** No recombination, **(B, D, F)** recombination, **(A, B)** sparse initial affinity, **(C, D)** minimal initial affinity, **(E, F)** moderate initial affinity. All populations were evolved with high mutation rates.

**Supplementary Figure S16. Complexity trajectories for simulations with modified mutation effect distributions.** Complexity (y-axis) shown as mean ± SEM (n = 10 replicates). Populations were evolved with mutation effect sizes sampled from varying Gaussian distributions; σ = 0.5 (teal), σ = 1.0 (blue), σ = 2.00 (black; value used in main simulations) and σ = 4.0 (red). Panels show: **(A, C, E)** No recombination, **(B, D, F)** recombination, **(A, B)** sparse initial affinity, **(C, D)** minimal initial affinity, **(E, F)** moderate initial affinity. All populations were evolved with high mutations rates.
